# Corneal parameters 18 Months following collagen cross-linkage treatment (CXL) for keratoconus in western Saudi Arabia: A prospective cohort study

**DOI:** 10.1016/j.amsu.2020.09.010

**Published:** 2020-09-09

**Authors:** Ashjan Bamahfouz, Serene Jouhargy, Ahmed Basheikh, Nawaf Alqahtani, Yaser Elhams, Ayat Aldosari, Faisal Khattab, Ensa Alkhoutani, Khadija Alfaqih

**Affiliations:** a Department of Ophthalmology, Umm Al-Qura University, Mecca, Saudi Arabia; b Department of Ophthalmology, Basafar Eye Centre, Jeddah, Saudi Arabia; c Department of Ophthalmology, King Abdulaziz University, Jeddah, Saudi Arabia; d Department of Ophthalmology, King Abdullah Medical City, Mecca, Saudi Arabia

**Keywords:** Keratoconus, Corneal dystrophies, Corneal ectasia, Collagen cross-linkage, CXL

## Abstract

**Introduction:**

keratoconus is a common disease in the general population, with prevalence ranging up to 200 per 100 000 with a reported increase in Saudi Arabia. Collagen Cross-Linkage is now an established treatment in isolation and in conjunction with other modalities for managing keratoconus. Our aim is to evaluate using a cohort study the impact of the treatment over a course of 18 months.

**Methods:**

To evaluate the impact of 18 months after collagen cross-linkage treatment and its determinants in eyes with keratoconus in Western Saudi Arabia. A one-armed prospective cohort study design on 45 patients with Stage I, II, III and IV keratoconus who were treated by Collagen Cross-Linkage modality was developed at our institute between 2018 and 2019 to establish the success rate of corneal ectasia stabilization of the disease.

**Results:**

Demographic data and grades of keratoconus (Amsler - Krumiech classification) at presentation were correlated to changes in corneal parameters 18 months after CXL compared to that at presentation. Stage I, II, III and IV keratoconus were 13, 14, 2 and 16 eyes respectively. The study showed that the K max significantly declined (P = 0.05) while spherical equivalent refractive status changed from median −1.5D to −2.27D (P = 0.002). Meanwhile, Central corneal thickness significantly reduced (P = 0.001).

**Conclusion:**

CXL can prove to be efficient in the treatment of Keratoconus and more studies should study ways to improve and implement this treatment plan to such patients.

## Highlights

• Keratoconus prevalence has been increasing in Saudi Arabia and the world.
• Studying treatment procedures of the disease is important to determine the best course of action.
• Collagen Cross-Linkage Treatment is a treatment option that showed variation of success rate in the literature.

## 1 Introduction

Keratoconus (KC) is a progressive, bilateral, asymmetrical corneal degeneration. KC is a common disease in the general population, with prevalence rate of 200 per 100 000. Twenty percent of keratoconus patients present with irregular astigmatism leading to severe visual deterioration [1]. The inciting mechanism for keratoconus remains unknown, however genetic and environmental factors have been implicated. Environmental factors include excessive eye rubbing, allergy and sun exposure which can lead to greater expression of reactive oxygen species [2]. Nonetheless, Collagen cross-linkage (CXL) is now an established treatment in isolation and in conjunction with other modalities of managing keratoconus [3,4].

Even though Keratoconus is highly prevalent in Saudi Arabia [5], CXL is now becoming an established procedure in different eye institutions in Saudi Arabia with promising outcomes [6,7]. Meanwhile, the earlier publications from central, eastern and western cities of the Kingdom were followed up for 12 months. Therefore, a study about outcome of collagen cross-linkage was sought to shed more light on the disease outcome. To the best of our knowledge, this is the no published studies to assess the outcome of collagen cross-linkage treatment from Macca city of western Saudi Arabia with a long term follow up of more than 12 months. For that, we present outcomes of this prospective study of collagen cross-linkage treatment and its determinants after 18 months in eyes with keratoconus at Macca, Saudi Arabia.

## 2 Materials and methods

This study was approved by the institutional review board of King Abdulla Medical City, Saudi Arabia registered at the National Biomedical Ethics Committee, King Abdulaziz City for Science and Technology and was registered in the Research Registry with ID: researchregistry5660. All keratoconus patients treated with CXL between June 2017 and March 2018 were invited to participate in this study. After their informed written consent, they were included in the study. Their identity was delinked from the data prior to analysis to maintain confidentiality. An eye with central corneal thickness less than 400 μ was excluded for CXL and present study as well as patients who underwent other corneal surgical procedures in the eye to be treated by CXL were also excluded. Afterwards, the rest of patients diagnosed with Keratoconus were then included.

For a one-armed cohort, we assumed that K_max_ improves in three forth of eyes treated with CXL. To achieve a 95% confidence interval and 80% power in the one-armed cohort, we need to study at least 41 eyes with keratoconus. To compensate for the loss of participants in 18 months of follow up, we increased the sample to 45. We used OpenEpi software to calculate the sample size [8].

Three cornea surgeons managed these cases with minimum 10 years of experience. The demographic data included the age and gender of the participant. In the case of bilateral keratoconus, the eye planned for the 1st CXL procedure was included in the study.

Distance vision was assessed using a projection chart to document the best-corrected distance visual acuity (BCVA) using pinhole and documented in decimal notation. Moreover, K_max_ and the central corneal thickness (CCT) was measured using Pentacam tomographer (Oculus Optikgeräte GmbH, Wetzlar, Germany). The refraction was carried out using cycloplegic refraction using 0.5% tropicamide eye drops. To calculate spherical equivalent, we used formula sphere + (cylinder/2).

Corneal topography was done using OPD Scan III (NIDEK Co. Ltd., Gamagori, Japan). Based on K_max_, the keratoconus was graded using the Amsler-Krumiech classification [9]. Eyes with KC were grouped into Stage I (K ≤ 48.0D), Stage II (48.1–53.0D), Stage III (53.1–55D) and Stage IV (55.1D & +).

The details of the CXL applications that was used (Dresden protocol) in the present study are described in details in the literature [10]. All patients underwent the epi-off technique. The eye was prepared in a sterile fashion and topical anesthesia was instilled in the operative eye and a lid speculum was inserted. The epithelium was removed by mechanical debridement using a number 64 blade. Riboflavin drops were instilled on the cornea every 2 min over 30 min. The eye was then exposed to ultraviolet-A light for 3 min. A bandage contact lens was placed on the cornea. Postoperatively, the patients were prescribed topical antibiotics for one week as well as a tapered schedule of a topical steroid and topical lubricants four times a day for two months or longer as needed.

The data was collected using a pretested data collection form. Data was filtered and transferred into a spreadsheet of statistical package for social studies; SPSS V 22.0 (IBM Corp., Armonk, NY, USA). Numerical data are presented as mean, median, standard deviation with interquartile range (IQR). Preoperative data were compared to postoperative data using the Wilcoxon Signed Ranks Test. All alpha values were 2 sided and a P value less than 0.05 was considered statistically significant. This study has been reported in accordance to the STROCSS guidelines [11].

## 3 Results

**Fig. 1:**
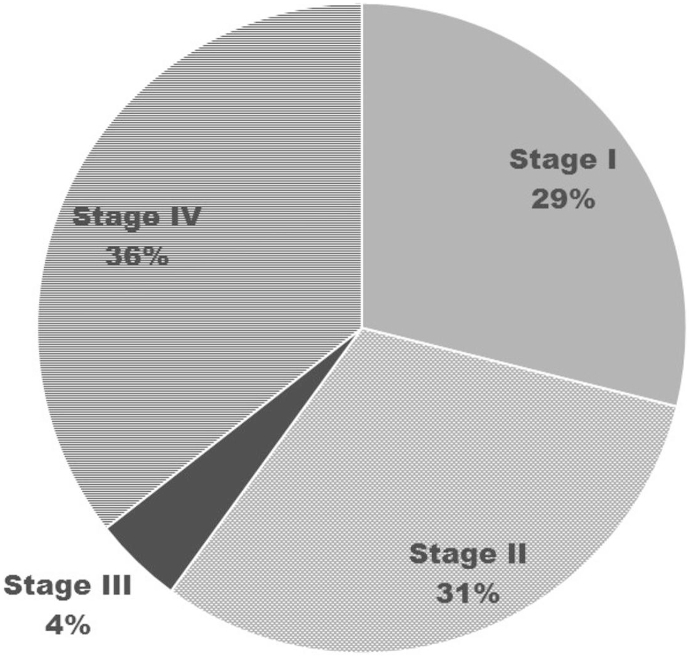
Proportion of eyes by grade of keratoconus.

Our cohort included 45 eyes of 45 patients with keratoconus with 6 cases lost in follow up decreasing it to a total of 39 patients. The mean age was 25 ± 5.3 years with males mean age of 25 (55.6%), right eye (22; 48.9%) and 38 eyes had unilateral KC. Moreover, regarding the grades of KC apart from stage III, all other stages of KC were equally distributed (Fig. 1).

The corneal parameters, refractive status and visual acuity at presentation and 18 months after CXL treatment is compared in Table 1. The K _max_ significantly declined (Wilcoxon P = 0.05). The success of stabilizing keratoconus was achieved in 29/39 = 74.4% (95% CI 60.7; 88.1). If all missing 6 cases on the follow up were stable, then the success rate would be 77.8%. But if all 6 missing cases were assumed to have deterioration of K_max_, then the success rate would be 64.4%.

**Table: 1:** Changes 18 months after and before Collagen cross linkage treatment (CXL).

		Before CXL	18 months after CXL	Validation (Wilcoxon P).
K_Max_	Mean	52.315	51.604	0.05*
	Median	50.5	49.7	
	Standard Deviation	±6.6	±6.2	
	IQR	47.6; 57.6	47.4; 56.7	
CCT	Mean	497	488.33	0.001*
	Median	499	499	
	Standard Deviation	±26.05	±43.98	
	IQR	447; 497	443; 487	
BCVA	Mean	0.8	0.83	0.9
	Median	1	1	
	Standard Deviation	±0.34	±0.28	
	IQR	0.45; 1.0	0.5; 1.0	
Spherical equivalent	Mean	−2.4853	−3.4740	0.002*
	Median	−1.5	−2.2750	
	Standard Deviation	±3.2	±6.25	
	IQR	−4.9; −0.4	−6.25; −0.19	

IQR = Inter Quartile Range; CCT = Central corneal thickness; BCVA = Best corrected visual acuity.

We associated the success to stabilize corneal curvature 18 months after CXL to the grade of keratoconus. The success of stabilizing the corneal curvature in eyes with keratoconus at 18 months after CXL was not significantly different by the grade of keratoconus at presentation (Table 2). The change in the corneal parameter in each eye at 18 months compared to before CXL was estimated and was reviewed in a cohort of cases with KC. Table 3. CCT declined in the majority of eyes. Refractive status shifted to myopia side but less than -1D. BCVA remained the same. K_max_ had marginally improved.

**Table: 2:** Decline in parameter among eye with keratoconus before & 18 months after CXL.

		Difference of parameter
K_Max_	Number	39
	Mean	−0.6
	Median	−0.2
	Standard Deviation	±3.34
	IQR	−1.3; 0.4
CCT	Number	39
	Mean	−11.7
	Median	−9.5
	Standard Deviation	±15.48
	IQR	- 25.8; 0.0
BCVA	Number	39
	Mean	−0.02
	Median	0.0
	Standard Deviation	±0.25
	IQR	−0.1; 0.1
Spherical equivalent	Number	39
	Mean	−2.02
	Median	−1.1
	Standard Deviation	±7.2
	IQR	−1.7; 0.25

CCT = Central corneal thickness; BCVA = Best corrected visual acuity.

**Table: 3:** Stabilization of keratoconus 18 months after collagen cross linkage by the stage of keratoconus.

Keratoconus stage based on K_max_ before CXL	Improved/stable		Progression		Validation
	Number	%	Number	%	
Stage I (N = 11)	9	81.8	2	18.2	χ 2 = 2.2 Df = 4 P = 0.14
Stage II (N = 11)	9	81.8	2	18.2	
Stage III (N = 2)	2	100	0	0.0	
Stage IV (N = 15)	9	60	6	40	
Total (Missing = 6)	29	74.4	10	25.6	

There were 16 of 22 male keratoconus patients with stable corneal curvature. There were 13 of 20 females with stable corneal curvature. Information of three cases was missing. Stabilization of keratoconus was not associated to gender (P = 0.6). Meanwhile, there were 23 out of 32 unilateral KC cases with stable corneal curvature. Furthermore, there were 13 out of 17 bilateral KC cases with stable corneal curvature. Lastly, the stabilization of keratoconus was not associated with gender (P = 0.8).

## 4 Discussion

In this cohort, the keratoconus cases of different grades showed stabilization of corneal curvature in nearly three fourth of eyes treated with CXL before 18 months. The corneal thickness marginally but significantly decreased, faced significant myopic shift and no change in corrected visual acuity. The stabilization was similar in all grades of keratoconus. It was not different in both genders and by laterality.

The adult Saudi population in the Macca region is 573 750 in 2017 [12]. If the prevalence of 5.6% as noted by Netto et al. [5] is applied to this population, there could be as many as 32 000 persons suffering from keratoconus. These cases need early detection and prevention measures to halt progression and keratoplasty surgeries. CXL to these identified cases shows promising results in the present study to have long term stabilization of cornea and thus delay in the progression of keratoconus. Sandvik et al. [13] reported a 47% decrease in corneal transplant procedures for keratoconus that they attributed directly to the introduction of CXL. Thus, the present study in the Macca region demonstrates the benefits of CXL.

The reduction of K_max_ a proxy indicator of the stable cornea following CXL in the present study was significant by hardly 0.6D. This matched with findings of [14]. But our findings are much less than 2D reduction noted in the literature [15,16]. The former having preliminary results while later had 12 months follow up. Therefore, comparison should be done with caution. Concerns have been raised regarding the repeatability of corneal measurement in the eye with corneal ectasia [17]. Perhaps, this could explain the differences coming in variation in success rates in studies conducted in different subcontinents and in a different time period.

In our study, there was a marginal myopic shift 18 months following CXL. This was similar to <1D reported by Jankoy et al. [18]. With 4 years follow up, Wollensak et al. [10] reported more than 1D shift. These data demonstrate the need for simultaneous refractive surgeries to correct the residual refractive error if needed [19]. In the literature, axial length has been reported to be linked with increased age [20,21]. However, in our study, as the mean age of the patients was 25 ± 5.3, it is believed the axial length was not related to age in this particular matter.

The best-corrected visual acuity in eyes treated with CXL for KC was similar to before CXL. This matches with the marginal myopic shift of RE. It also reflects a minimum corneal haze. Although in the present study, we did not document the presence and extent of corneal haze at the last follow up. Corneal haze is noted as transient complication more pronounced in eyes with advanced grade of keratoconus [22]. With 18 months of follow up, perhaps this corneal haze has subsided resulting in it not significantly affecting distance vision. A study showing 10 years follow up of CXL in children showed improvement if Corrected distance visual acuity by 0.14 logMAR and in another study in adult there was significant improvement in BCVA [23,24]. The best corrected visual acuity does not directly reflect functional outcomes, as it is linked to refractive error status, complications of intervention such as corneal haze and inflammatory process in anterior segment of eye due to CXL. Therefore, prognosis should not be promised to patients based on vision improvement.

We did not find any significant association of success in flattening corneal curvature to the grade of keratoconus. In most of the studies evaluating the impact of CXL, grading of keratoconus was used for inclusion criteria [15,19]. Mofty et al. [25] studied the grades of keratoconus but had the inclusion of corneal thickness for grading and suggested poor outcomes in very steep corneas. In a review of keratoconus protocols, the authors mentioned Amsler-Krumiech classification based grouping but had also grading by type of conventional vs accelerated method of exposure [26]. The association of grade of keratoconus to outcome in our study should be interpreted with caution as the sample size was not calculated to study this association.

There were a few limitations to our study. One-armed cohort could reflect medium-term follow-up results. These KC patient's short-term outcomes were not documented. The duration of follow up in different studies is varying. These were also highlighted in a Cochrane review [27].

## 5 Conclusions

Collagen cross-linkage in a country where the magnitude of keratoconus is on rising and availability of donor cornea is limited, CXL will be the choice of intervention at many institutions. These institutions with small samples may not be able to interpret the outcomes so as to propose a revision of protocol to improve outcome and therefore meta-analysis is recommended. Steep cornea seems to be a good indicator to judge the success of flattening the cornea by subjecting a KC eye with CXL. However, CCT should be monitored for a long time to ensure further things may not result in sign threatening complications. Cornea surgeons if trained in undertaking concurrent refractive surgery in the KC eye, both progression can be stopped, and refractive status can also be improved.

## Ethical approval

The IRB of King Abdulaziz City for Science and Technology.

## Author contribution

All authors were part of designing, data collection, writing, analysis, proofreading and referencing of this study.

## Registration of research studies

1. Name of the registry: Research Registry.

2. Unique Identifying number or registration ID: 5660.

3. Hyperlink to your specific registration (must be publicly accessible and will be checked): https://www.researchregistry.com/browse-the-registry#home/registrationdetails/5ed3a786cd312c001519d8d8/.

## Guarantor

Ashjan Bamahfouz.

Ashjanmd@gmail.com.

## Consent

Ethical approval and consent taken.

## Funding

None.

## Provenance and peer review

Not commissioned, externally peer reviewed.

## Declaration of competing interest

The authors declare no conflict of interest.
